# Case report: Analysis of a case of hydrogen sulfide poisoning in a waste treatment plant

**DOI:** 10.3389/fpubh.2023.1226282

**Published:** 2023-10-27

**Authors:** Aerbusili Genjiafu, Mengdi Shi, Xiangxing Zhang, Xiangdong Jian

**Affiliations:** ^1^School of Public Health, Cheeloo College of Medicine, Shandong University, Jinan, China; ^2^Department of Poisoning and Occupational Diseases, Emergency Medicine, Qilu Hospital of Shandong University, Cheeloo College of Medicine, Shandong University, Jinan, China

**Keywords:** waste treatment plant, hydrogen sulfide poisoning, sludge treatment tank, analysis of the case, self-protection

## Abstract

This paper summarizes and analyzes the clinical data of a patient with Occupational hydrogen sulfide poisoning admitted to our hospital on March 4, 2023. On the morning of March 2, 2023, the patient worked at an environmental energy company (waste treatment plant) in Shandong Province for the first time, The job was to flush the sludge from the walls of the sludge treatment tank (anaerobic tank) with a water gun, which can release hydrogen sulfide gas. When the patient was about to start work after entering the tank for about 1 min, he suddenly smelled a harsh and pungent odor, felt dizzy and weak, and then the patient suddenly fainted. After hearing the sound of the patient fainting, the workman waiting at the entrance of the tank immediately called someone to go into the tank and quickly pull the patient out, and sent to the local hospital. In the local hospital, the patient was confused, accompanied by irritability, convulsion and other manifestations, and was treated with sedation and nutritional support. Two days later, the patient’s condition did not improve. For further diagnosis and treatment, the patient was transferred to the Department of Poisoning and Occupational Diseases in our hospital. After comprehensive treatment in our hospital, the patient got better and was discharged. Subsequent reexamination and follow-up showed that the patient recovered well. The work unit of the patient did not provide any personal protective equipment. According to the field investigation after the accident, the pipeline around the sludge treatment tank was blocked by sludge, resulting in a large amount of high concentration of H_2_S accumulated in the tank, causing the patient to faint soon after entering the tank, and his worker should be in the tank for a short time, and no health abnormalities were found. Hydrogen sulfide has a strong irritation to the human body, which can lead to asphyxia or even death in severe cases. The safety prevention and prevention knowledge of hydrogen sulfide poisoning should be popularized among enterprises and workers to reduce the occurrence of such incidents.

## 1. Introduction

Hydrogen sulfide, whose chemical formula is H_2_S, is one of the major toxic gases (1), and has the characteristic odor of rotten eggs (2). H_2_S can be found in the oil and gas industries. It is used in activities such as food processing, paper mills, and tanneries. H_2_S could damage one’s eyes, respiratory system and central nervous system when inhaled at a low concentration (3). However, it could cause permanent brain damage at a higher concentration, even death because of a respiratory failure (4, 5). It is the most common cause of occupational gas exposure deaths rank only second to carbon monoxide, particularly in the oil and gas, sanitation and so on (6, 7). The production speed of H_2_S is very fast, and in a closed environment, such as a car or a small room, it can reach a fatal concentration (7). H_2_S poisoning is usually related to the operation accidents in the confined space. That is because, in the confined space, due to the limited into the hole and adverse natural ventilation, produce poisonous gas material will be the breakdown and fermentation processes (8). Environmental Protection Agency’s (EPA) toxicological review of hydrogen sulfide reported that multiple occupational exposures led to rapid toxicity and sudden but reversible loss of consciousness, which was called “knockout” (9). At present, there is no antidote for sulfide poisoning, and the treatment is supportive to a great extent (10). No matter the severity of the initial coma, there were no obvious long-term adverse reactions among the survivors (11).

## 2. Case description

**Figure 1:**
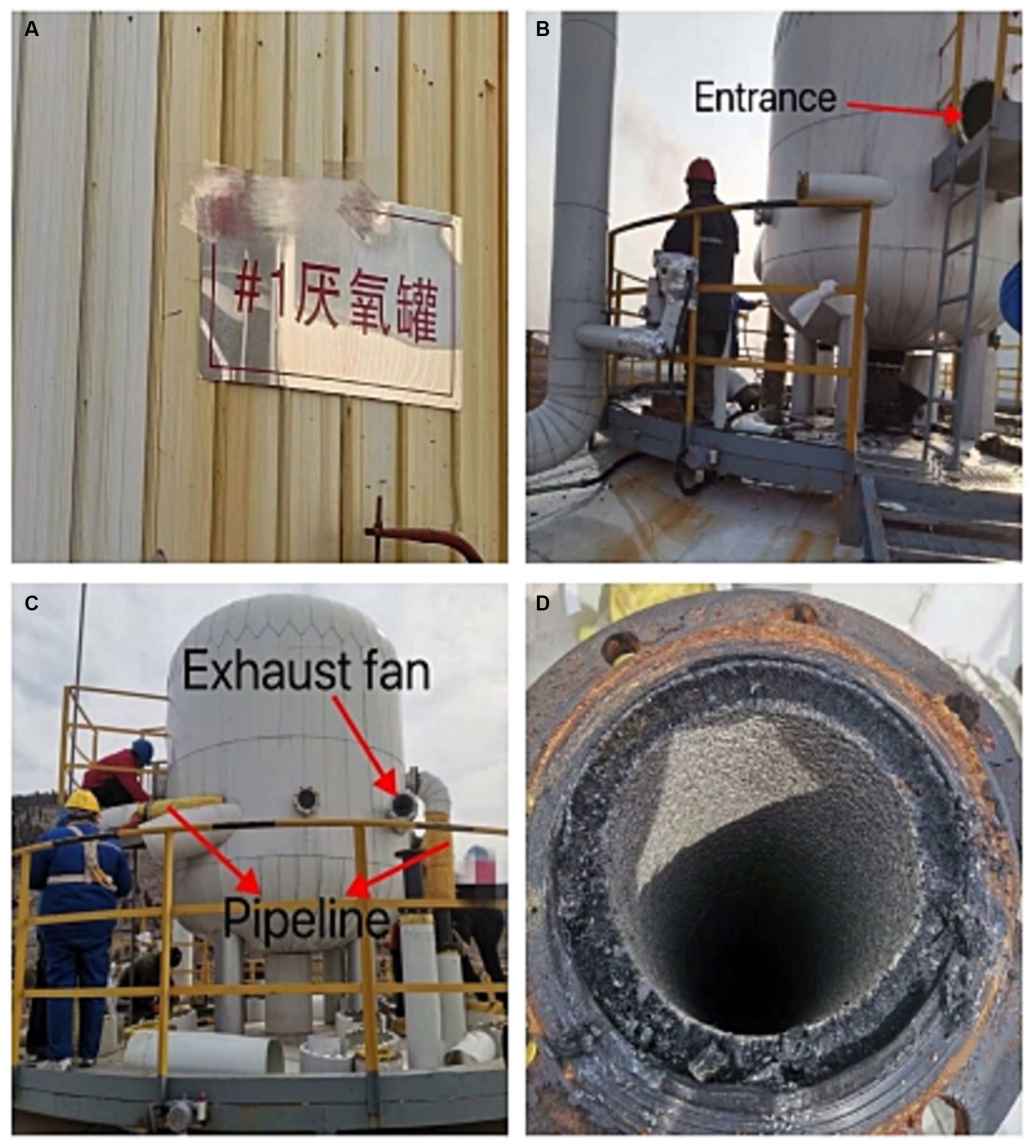
**(A)** Signs in Chinese for anaerobic tanks. **(B,C)** Exterior view of sludge treatment tank. **(D)** Interior view of sludge treatment tank.

**Figure 2:**
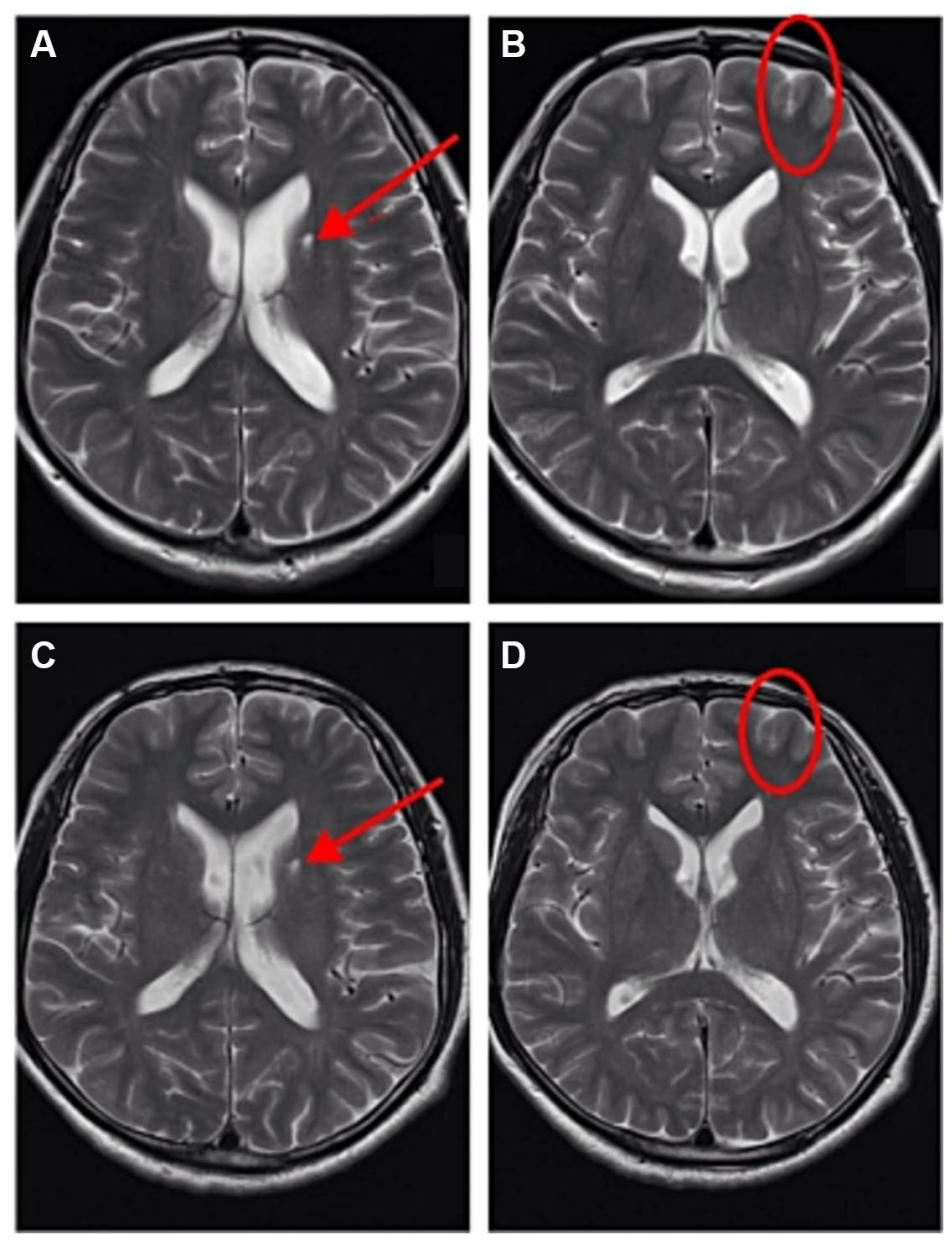
The patient underwent Brain MRI on 2023/3/6 **(A,B)** and 2023/3/26 **(C,D)**. **(A)** There were abnormal signals in the left basal ganglia, considering softening foci. **(B)** There were a few abnormal signals in the left frontal lobe, considering non-specific signals or degenerative lesions around the perivascular space. **(C)** There were still abnormal signals in the left basal ganglia, considering softening foci, but the extent of the lesion was reduced compared with **(A)**. **(D)** There were still a few abnormal signals in the left frontal lobe, considering non-specific signals or degenerative lesions around the perivascular space, but the extent of the lesion was reduced compared with **(B)**.

**Figure 3:**
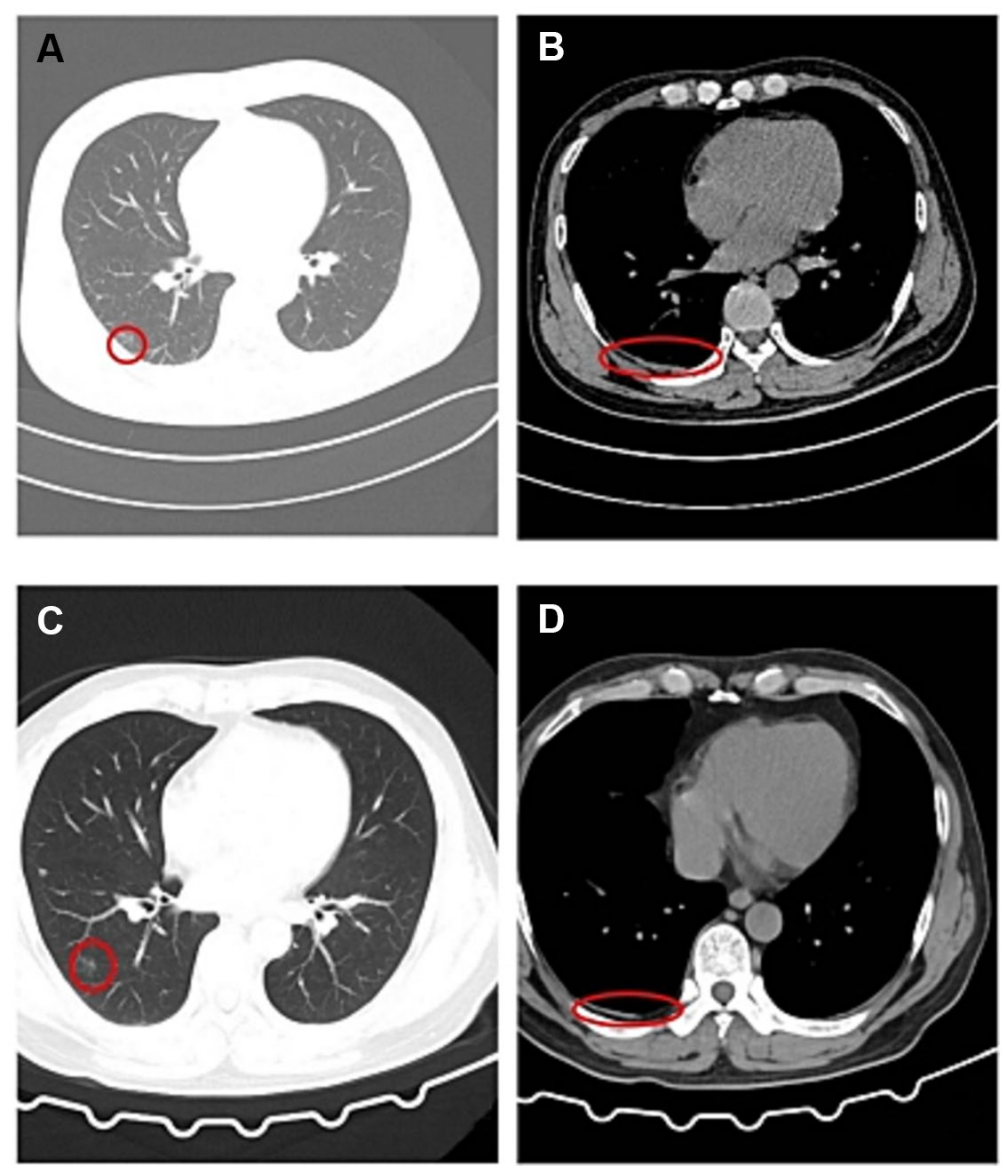
The patient underwent Chest CT on 2023/3/6 **(A,B)** and 2023/3/26 **(C,D)**. **(A)** There was a little exudation in the lower lobe of the right lung, with localized interstitial changes. **(B)** There was localized thickening of the right pleura. **(C)** There was still exudation in the lower lobe of the right lung, but the extent of the lesion was reduced compared with **(A)**. **(D)** There was still localized thickening of the right pleura, but the extent of the lesion was reduced compared with **(B)**.

The patient was a male, 46 years old. On the morning of March 2, 2023, he worked in an environmental protection energy company in Shandong Province for the first time, which was a waste treatment power plant. His job was to flush the sludge from the inner wall of the sludge treatment tank (Figure 1, anaerobic tank, 4 m high, 3 m diameter) with a water gun, and the sludge could release hydrogen sulfide gas. According to his and his co-workers, the patient was about to start work after entering the tank for about 1 min, when he suddenly smelt a harsh, pungency smell, and felt dizzy and weak, then the patient suddenly fainted. After hearing the sound of his fainting, the workers waiting at the entrance of the tank immediately called someone to enter the tank and quickly pulled him out, and then sent to the local hospital. At the local hospital, the patient was confused, accompanied by irritability, convulsions and other manifestations, and was treated with sedation (Midazolam and Diprivan) and nutritional support [Structured fat emulsion (20%)/amino acid (16%), glucose injection (13%)]. Two days later (2023.3.4), the patient’s condition did not improve. For further diagnosis and treatment, the patient was transferred to the Department of Poisoning and Occupational Diseases in our hospital. The patient awoke in the ambulance while being transferred to this hospital with gradual improvement in consciousness but no recollection of the events that followed. After investigation, the causes of this accident were as follows: the labor unit of the patient did not provide any personal protective equipment, and the patient only wore an ordinary raincoat into the tank; The pipeline around the sludge treatment tank was blocked by sludge, resulting in the accumulation of a large number of high-concentration hydrogen sulfide gas in the tank, causing the patient to faint soon after entering the tank. Although there is an exhaust fan on the tank wall, the volume of the exhaust fan is too small to have any effect. Due to the very short time in the tank, the worker pulled out the patient immediately after entering the tank, and no health abnormalities were found. He was admitted to our hospital with a “disturbance of consciousness for 2 days,” and physical examination findings were as follows: temperature was 37°C; pulse was 95 times/min; respiratory rate was 18 times/min; blood pressure was 131/79 mmHg; and SPO_2_ was 96%. The patient had blurred consciousness, poor spirit, and autonomous posture; he was cooperative during cooperation. The skin mucosa of the whole body was not yellow, and the superficial lymph nodes were not large. His bilateral pupils were large and round, reflecting light, and the size of the pupil was 3 mm. There was no cyanosis or congestion in the pharynx. The patient had a soft neck with no resistance, bilateral thorax movement, clear lung sounds when breathing, and no dry and wet rales. The rhythm was normal and there was no pathological murmur in the valve area. The abdomen is soft, there is no tenderness and rebound pain in the whole abdomen, liver, spleen, and ribs, and there is no tapping pain in the liver and kidney areas. No deformity of spine and limbs. The patient had physiological reflex and no pathological reflex. The results of the patient’s blood draw after admission from 2023/3/6 to 2023/3/26, as shown in Table 1, were basically normal except for a higher number of inflammatory cells (neutrophils ratio was high on Day 1 and Day 3, and the total number of white blood cells was high on Day 1), which indicated an inflammatory response in this patient. Magnetic resonance imaging (MRI) after admission (2023/3/6), as shown in Figure 2, patchy long TI and long T2 signal shadows were seen in the left basal ganglia, and T2 FLAIR showed medium-low peripheral hyperintensity with clear boundaries. The left frontal lobe showed a speckle-like long TI and long T2 signal shadow, T2 FLAIR hyperintensity, and DWI isointensity. The examination concluded that abnormal signals in the left basal ganglia, considering softening foci (Figure 2A); There were a few abnormal signals in the left frontal lobe, considering non-specific signals or degenerative lesions around the perivascular space (Figure 2B). And the subsequent reexamination on 2023/3/26 revealed a slight reduction in the extent of the lesion (Figures 2C,D). On CT on 2023/3/6, as shown in Figure 3, there was a little exudation in the lower lobe of the right lung, with localized interstitial changes (Figure 3A), which were relieved on the second examination on 2023/3/26 (Figure 3C). There was localized thickening of the right pleura (Figure 3B), which were also relieved on the second examination (Figure 3D). According to the diagnostic criteria of occupational acute hydrogen sulfide poisoning of the People’s Republic of China (12), the patient had a history of exposure to a large amount of inhaled hydrogen sulfide in a short period of time, and the clinical manifestations of the central nervous system and respiratory system appeared. The on-site labor hygiene investigation also supported the above exposure history, so the patient was diagnosed with occupational acute hydrogen sulfide poisoning. As a consequence, the following management regimen was applied. Dexamethasone (10 mg, ivdrip, qd) was given to reduce the inflammatory reaction, and the drug was discontinued on the 4th day. Salvianolate (200 mg, ivdrip, qd) to improve microcirculation; Torasemide (20 mg, iv, bid) to reduce edema and speed up the excretion of poison; Nalmefene (0.1 mg, iv, bid) to alleviate the symptoms of consciousness disorder and promoting the recovery of consciousness in the patient and was discontinued on Day 3. Flucloxacillin (1 g, ivdrip, q6h) for infection prevention; In addition, he was treated with sedation (Midazolam and Diprivan) and nutritional support [Structured fat emulsion (20%)/amino acid (16%), glucose injection (13%)]. After comprehensive treatment in our hospital, the patient got better and was discharged on the 7th day of admission (2023/3/10), and then came for reexamination on 2023/3/26. Studies involving human subjects were reviewed and approved by the Ethics Committee of Qilu Hospital of Shandong University. The patient provided written informed consent to participate in this study. Personal written informed consent has been obtained for the release of any potentially identifiable images or data contained in this article.

**Table 1:** Results of the laboratory examinations of the patient.

Admission time	Day 1	Day 3	Day 7	Day 21	Reference value
WBC (10^9^/L)	6.53	11.57	9.26	8.00	3.50–9.50
NEU (%)	91.60	83.10	65.90	65.00	40.00–75.00
TP (g/L)	79.0	67.6	57.0	69.6	60.0–85.0
ALB (g/L)	43.0	41.5	35.7	43.6	40.0–55.0
ALT (IU/L)	41	48	71	36	9–50
AST (IU/L)	37	21	27	23	15–40
BUN (mmol/L)	9.60	10.00	7.60	4.40	2.30–7.80
Cr (μmol/L)	76	71	67	71	62–115

WBC, White blood cells; NEU, Neutrophils; TP, Total protein; ALB, Albumin; ALT, Alanine aminotransferase; AST, Aspartate aminotransferase; BUN, Urea nitrogen; Cr, Serum creatinine.

## 3. Discussion

H_2_S is poisonous, and accidents may occur on exposure to natural gas, volcanic gas, and industrial waste (13), this patient was also poisoned after exposure to industrial sludge. Accidents have been reported in chemical processing plants (14, 15) and sewage disposal facilities (16–19) and with the ingestion of sulfur products (20, 21). The same as reported by Jack et al. (16–19), This patient’s H_2_S poisoning accident occurred at a sewage disposal facility. The acute toxicity of H_2_S mainly involves the central nervous system and lungs (22), which is consistent with this patient’s clinical presentation. The oxidation of H_2_S after exogenous exposure is not complete, and the remaining H_2_S *in vivo* exists in a dissolved and bound form (23). The dissolved forms are H_2_S gas and sulfhydryl anion; both forms can diffuse between blood and tissue. The dissolved forms are H_2_S gas and sulfhydryl anion; both forms can diffuse between blood and tissue (24). Bound, insoluble sulfide forms include acid-labile sulfides and sulfated proteins (23). Both forms capture sulfide in an insoluble state and play a role in toxicity (23). For example, sulfide can bind to the iron atoms of the electron transport chain complex IV to inhibit cytochrome c oxidase (2, 25). Jiang et al. (10) described the direct toxicity to neurons derived from human induced pluripotent stem cells, leading to apoptosis. Cellular damage by reactive oxygen species, such as the increase in protein kinase (JNK and Erk) activity and the production of f2-isoprostane (a prostaglandin-like compound as a marker of oxidative stress), occurs after exposure to sulfide (26). Exposure to high concentrations of H_2_S can cause various neurological symptoms, such as dizziness, headache, poor coordination, and transient loss of consciousness. If exposure is transient, recovery is usually complete and rapid (27). A unique feature of this toxic gas is that sudden exposure to high concentration will lead to “knocking down” (28). This is a kind of incapacitating influence, which makes the victim unable to escape (29). For the same reason with Wang et al. (27), Anantharam et al. (28), and Guidotti et al. (29), the patient fainted after being exposed to high concentration of H_2_S and stayed in an anaerobic tank with poor ventilation for some time without personal protective equipment, resulting in H_2_S poisoning. Fortunately, after the accident, his workmates were able to find and rescue the patient in time, and immediately sent him to the hospital for treatment. The final result was that the patient recovered quickly without affecting the quality of life of the patient. The most frequently involved site in the brain is bilateral basal ganglia nuclei; because these nuclei need a lot of oxygen, the imaging features are symmetrical low density/high signal around bilateral basal ganglia and lateral ventricle; The lesions were distributed in bilateral frontoparietal white matter, oval center, lateral ventricle periphery, and basal ganglia nuclei (30). In patients with severe clinical symptoms, brain CT/MRI showed all four signs (systemic brain edema, symmetrical low density/abnormal signal around bilateral basal ganglia and lateral ventricle, subarachnoid hemorrhage or intracerebral hemorrhage, and cerebellar tonsil hernia) within 2 months; Systemic brain edema and symmetrical low density/abnormal signals around bilateral basal ganglia and lateral ventricles are the main findings (30). In contrast to the findings reported by Tang et al. (30), the imaging findings in this patient were unilateral rather than bilateral, probably because of the less severe degree of poisoning in this patient, which resulted in an atypical lesion. Lancia et al. (31) reported a fatal case of a factory worker who died after breathing H_2_S while performing a job for which he was not trained. Goolam et al. (32) described a 30-year-old sewer worker with a history of severe unexpected occupational exposure to inhaled H_2_S, and ultimately death due to acute respiratory distress syndrome and rapid respiratory depression. Sheikh et al. (33) reported a case of a fisherman exposed to a high concentration of H_2_S in a fish garbage room, and eventually, the patient survived and was discharged with a full recovery. The case we reported was clinically cured. Through telephone follow-up, we learned that the patient had no health abnormalities and has been transferred from his original post. However, this paper also has limitations, we did not obtain measurement data of H_2_S in the blood, nor did we obtain data of H_2_S in the field environment. We will continue to improve our work in the future.

Based on this accident, I have gained the following enlightenment to prevent occupational poisoning. The government and employers should work together to provide a safe and comfortable working environment for workers. It is suggested to reinforce the government’s responsibility for occupational disease prevention and control the occurrence of occupational disease hazards from the source. Establish an effective supervision mechanism for occupational disease prevention departments and an evaluation system for occupational disease prevention; Enhance occupational health supervision and law enforcement; Carry out in-depth publicity and training on occupational health laws and knowledge, give full play to the role of media publicity and public opinion guidance, continuously improve workers’ awareness of health protection and self-awareness of occupational disease prevention and control, and effectively prevent and control the occurrence of occupational diseases (34). Employers should adhere to the basic principle of people-oriented, always put the safety and health of workers in the first place, provide workers with a good working environment and personal protective equipment, strengthen the personal protection of workers, regularly carry out health education, carry out occupational health examination, eliminate potential safety hazards, and try to replace toxic or highly toxic substances with non-toxic or low-toxic substances. Implement occupational safety operation procedures. Workers should strengthen personal protection and work in accordance with occupational safety operating procedures.

## Data availability statement

The original contributions presented in the study are included in the article/supplementary material, further inquiries can be directed to the corresponding author.

## Ethics statement

Written informed consent was obtained from the individual(s) for the publication of any potentially identifiable images or data included in this article.

## Author contributions

AG and MS designed the study and wrote the manuscript. XZ drafted the manuscript. XJ revised the manuscript. All authors contributed to the article and approved the submitted version.
